# Latent Classes of Principals’ Transformational Leadership and the Organizational Climate of Kindergartens

**DOI:** 10.3389/fpsyg.2019.02015

**Published:** 2019-09-10

**Authors:** Pingping Wang, Xinrui Deng, Xiaowei Li, Yuan Dong, Runkai Jiao

**Affiliations:** ^1^School of Psychology, Northeast Normal University, Changchun, China; ^2^Faculty of Education, Beijing Normal University, Beijing, China

**Keywords:** kindergarten, principal, organizational climate, transformational leadership, latent class analysis

## Abstract

**Background:**

Organizational climate refers to an individual’s perception and experience of the climate of the work environment, and it is the most important environmental variable that affects individuals’ work performance. This study aims to classify characteristics of transformational leadership among kindergarten principals and examine their relationship to organizational climate.

**Methods:**

Convenience sampling yielded 498 kindergarten principals who completed the “Questionnaire on the Principal’s Transformational Leadership Behavior” and “Questionnaire on Organizational Climate.” Ethics approval was obtained from the Academic Ethics Committee of the College of Psychology of Northeast Normal University prior to starting the study.

**Results:**

Three latent classes were identified, including the high-level (68.8%), care-virtues (35.7%), and virtues groups (5.3%). There were significant differences in support, directive, restrictive, colleague, intimate, and disengaged behavior scores between groups. In terms of support, colleague, and intimate behavior, the high-level group had the highest scores, followed by the care-virtues group and virtues group, respectively. Regarding restrictive and disengaged behaviors, the highest scores were received by the virtues group, followed by the care-virtues and high-level group, respectively.

**Conclusion:**

The study suggested that principals’ transformational leadership could be classified into three latent classes that are related to organizational climate.

## Introduction

The global economic crisis that began in 2008 significantly affected workers worldwide, and its impacts continue in some countries (Mucci et al., 2016). The workers affected include kindergarten teachers. Workers’ anxiety concerning the economic crisis can lead to job stress and, thereby, to a decline in their mental health (Giorgi et al., 2015). However, a number of organizational characteristics may be significant in preventing and reducing the incidence of work-related stress (Giorgi et al., 2019), including organizational climate. As Snorradóttir et al. (2013) suggested, environmental factors are associated with psychological distress, and direct involvement in organizational and structural change is associated with the development of psychological distress. In the context of an economic crisis, transformational leaders are better able to adjust their organizational and management goals purposefully, adapt to the times, and create an ideal working atmosphere to maximize the performance of their employees.

### Organizational Climate

Organizational climate refers to an individual’s perception and experience of the climate of the work environment and is the most significant environmental variable that affects individuals’ work performance. In the context of a school, the organizational climate refers to the teachers’ perception of the school’s overall environment and ideology (Croft and Halpin, 1962; Dutta and Sahney, 2016). It is a persistent characteristic formed by the interaction between the principal’s behavior and the teachers’ behavior in the school environment (Cao, 2002). Extended to the specific context of kindergartens, the organizational climate is the unique atmosphere produced by the interaction between the director and the kindergarten teachers. Teachers directly or indirectly perceive events, activities, and procedures in their workplace (Litwin and Stringer, 1968; Chen, 2017). When these perceptions become a form of shared cognition among the kindergarten teachers, it becomes part of the organizational climate of the kindergarten. As the leader of the kindergarten, the organizational decision maker, and the creator of the organizational culture, the kindergarten director plays an important role in the formation of the organizational climate and the creation of a positive environment (Anggraini et al., 2018), which is important for teachers’ mental health. In this context, transformational leadership may have a profound impact on organizational climate.

### Transformational Leadership’s Impact on Organizational Climate

Research on the characteristics of leaders and organizational climate has shown that transformational leadership has a positive effect on the formation of an efficient, visionary, and mission-oriented organizational climate (Zuraik and Kelly, 2019). It can promote a culture of team innovation, create an atmosphere of equality and freedom, and promote cooperation among team members (Cantu, 2012; Liu, 2017). Transformational leadership is when leaders influence organizational members through their charisma and intrinsic qualities (Kohan and Safari, 2018) and provide an organizational vision that inspires employees to work enthusiastically, enhancing members’ motivation through sharing, stimulation, investment, and motivation (Zuraik and Kelly, 2019). Transformational leadership motivates followers to transcend their own interests for the benefit of the group (Yu et al., 2018). In the kindergarten context, transformational leadership is mainly manifested through the director’s instructions and advice that elicit expected responses by the teachers (Xiang, 2007), encouragement of teachers to establish a common vision (Rodd, 2007), establishment and realization of organizational goals, and effective development of the kindergarten (Liu, 2015). Moreover, transformational leadership and organizational climate can both impact organizational readiness for change (von Treuer et al., 2018).

Although few studies have examined transformational leadership in the context of kindergartens, research on schools has found a high correlation between transformational leadership and organizational climate (Swangi, 2016). Meta-analyses provide strong evidence of the positive impact of leadership on organizational climate (Karadağ et al., 2015). They also suggest that there is a close relationship between transformational leadership and improved employee attitudes (Wang et al., 2011) and work environments (Duan et al., 2017), a significant predictive effect of perceived supervisor support on organizational climate (Chen and Wang, 2015), and a close relationship between leadership behavior and organizational climate (Cai, 1985). Moreover, transformational leadership can further affect employee behavior *via* organizational climate (Ko and Kang, 2019). Based on these findings, it can be inferred that a director’s transformational leadership can effectively predict the characteristics of the organizational climate in kindergartens.

### The Present Study

In examining the relationship between transformational leadership and organizational climate, the results of variance-based research have reached a relatively consistent conclusion: the leadership levels of organizational directors can effectively predict the formation of a positive organizational climate. Leadership, the summation of leadership behaviors, must be considered in light of the correspondence between individuals and the organizational climate they create. Studies of leadership require an individual-centered approach that can distinguish the leadership levels of leader groups and identify the characteristics of the organizational climate that correspond to the leader’s Central Asian group. Category analysis techniques could establish a statistical model based on the relationship between explicit and potential category variables and be used to classify features of specific external manifestations of potential categories (Gao, 2018). Thus, this study aims to use latent class analysis (LCA) to explore the relationship between kindergarten directors’ transformational leadership and organizational climate from an individual-centered perspective, which may provide significant insight into this relationship. It is hypothesized that the transformational leadership of kindergarten principals can be categorized into several latent classes, and these classes have different influences on the organizational climate within kindergartens.

## Materials and Methods

### Participants

Six hundred questionnaires were issued, and 509 were collected; the questionnaire recovery rate was 85%. Eleven questionnaires were excluded due to incompleteness or extreme values, leaving a total of 498 valid questionnaires. The participants had normal visual acuity and no mental illness. Among the participants, 444 (89.2%) were women, and 40 (8%) were men; 45 (9%) were 30 years or younger, 183 (36.75%) were 31–40 years old, 240 (48.19%) were 41–50 years old, and 24 (4.82%) were older than 50 years, making the 41–50 age group the largest. Regarding education levels, 48 (9.6%) had completed high school or less, 406 (81.5%) had an undergraduate degree, and 17 (3.4%) had a master’s degree. There were 270 teachers from cities (54.2%) and 211 teachers from rural areas (42.4%). Fifty-seven (11.4%) principals had been working in this field for 5 years or less, 58 (11.6%) for 6–10 years, 152 (30.5%) for 11–20 years, and 222 (44.6%) for more than 20 years; 7 (1.4%) principals missed or misfiled this information. Regarding tenure in their current position, 114 (22.9%) principals’ tenure was no more than 2 years, 142 (28.5%) was 3–5 years, 106 (21.3%) was 6–10 years, and 111 (22.3%) was over 20 years; 25 (5%) principals missed or misfiled this information.

### Procedure

First, the study was approved by the Academic Ethics Committee of the College of Psychology of Northeast Normal University, and trained psychology graduate students served as the experimenters. After explaining the purpose and methods of the study to the participants, the questionnaires were distributed and collected with their consent. The questionnaires were tested in a random order. Considering the concentration required from the participants and to ensure the accuracy of the data, after completing one questionnaire, participants were given 5 min of rest before working on the other one.

### Measures

#### Questionnaire on the Principal’s Transformational Leadership Behavior

The questionnaire included 22 items addressing four dimensions: vision, charisma, individual consideration, and moral modeling (Ji, 2014). “Vision” addressed whether the principal and the teachers had established a common vision and goals and whether principals encouraged support for teachers. It included four items, such as “I can make teachers understand the direction of the kindergarten’s future development.” “Charisma” concerned the director’s ability to sway teachers with his or her own ability and wisdom and to use charm to encourage teachers to meet the organizational goals. It included five items, including “I love my job and has a strong sense of enterprise.” “Individual consideration” concerned the director’s ability to understand the needs of every teacher, to care for them, and to help them solve problems in their work and life. It included five items, including “I am willing to help teachers solve problems relating to their life and family.” “Moral modeling” addressed the director’s integrity, impartiality, and equality in working with teachers to promote the development of kindergartens. It included eight items, including “I don’t take credit for the achievements of teachers.” In this study, the α value was 0.913, and the correlation between each factor and total scores ranged from 0.796 to 0.87.

#### Questionnaire on Organizational Climate

The questionnaire included 40 items addressing six dimensions. “Support behavior” referred to the director’s support and care for teachers. It included seven items, such as “The director of the kindergarten relies on the teaching and management ability of the teacher.” “Directive behavior” items addressed whether the director’s leadership was task oriented and demonstrated little concern for employees and involved close supervision of employees and little delegation. It included nine items, including “Each specific duty within the kindergarten is assigned by the director.” “Restrictive behavior” referred to the principal’s tendency to require additional work from teachers that was not directly related to teaching. It included five items, such as “Kindergarten affairs often involve tedious procedures and forms.” “Collegial behavior” referred to the degree of appreciation among teachers and their willingness to cooperate and to discuss teaching-related challenges. It included nine items, such as “Teachers actively participate in on-the-job training in various fields.” “Intimate behavior” addressed the degree of friendship between teachers—the degree of mutual concern and care. It included five items, including “Teachers have close exchanges and care for each other.” “Disengaged behavior” referred to teachers’ sense that they did not belong in the kindergarten organization, that they maintained a certain distance from their colleagues and organization, and that they had no common organizational goals. It included five items, such as “Teachers often talk about being transferred to another post or departure.” For this questionnaire, the α value was 0.755, and the correlation between each factor and total scores was 0.576–0.831.

### Data Analysis

Most previous studies on principal leadership examined leadership levels based on a total score, but this approach is too broad and can yield inaccurate results. Classifying leadership through LCA can allow a more concrete and specific study of a group. This classification method, based on a probability model, not only ensures a maximum difference between categories and minimum difference within categories but also employs objective statistical indicators to measure the accuracy and effectiveness of classification. We used the Mplus 7.0 software to explore a potential category analysis of kindergarten director leadership and then to explore the types of kindergarten director leadership. Principals’ transformational leadership items were used as indicators of class membership. Several fit indices were used to determine the latent class structure, including the Bayesian information criterion (BIC), the bootstrapped likelihood ratio test (BLRT), and the Lo–Mendell–Rubin (LMR) likelihood ratio test (Lo, 2001). To interpret fit indices, the Akaike information criterion (AIC) and the BIC were used to indicate goodness of fit, with better-fitting models scoring lower; BLRT and LMR were used to compare models with different numbers of classes, and significant *p*-values suggest that a given model is preferable to a model with one less class. The entropy index was used to evaluate classification accuracy. When its value ranged between 0 and 1, the model was considered to have better predictability; when entropy = 0.6, it indicated that about 20% of individuals had classification errors. Entropy was also used to assess the overall degree of classification uncertainty, with values above 0.80 suggesting a greater distinction between classes (Celeux and Soromenho, 1996). Generally, if the entropy of a model is higher, the AIC, BIC, and aBIC (sample size-adjusted BIC) are lower, and the LMR and BLRT are significant, the model is considered to have a high degree of fit (Wang and Bi, 2018). In the current study, the best model was chosen by balancing parsimony, theoretical interpretability, and goodness of fit.

After latent classes were determined, we explored the relationships between principals’ latent class membership and the organizational climate of kindergartens. The differences in leadership types among directors in different organizational climates were analyzed using one-way analysis of variance in SPSS 21.0. The independent variable was the leadership type of the kindergarten principal identified through latent category analysis, and the dependent variables were each subdimension of the kindergarten organizational climate.

## Results

### Preliminary Statistical Analysis

#### The Transformational Leadership of Kindergarten Principals and Kindergarten Organizational Climate

Table 1 shows that the average value of transformational leadership in kindergartens was 4.431, the standard deviation was 0.410, and the average scores of the four leadership subdimensions ranged from 4.194 to 4.199, with moral modeling scoring the highest, followed by individual consideration, charisma, and vision, respectively. Table 1 also shows that the average value of the kindergarten organizational climate was 3.299, and the standard deviation was 0.246. The average score of the six organizational climate subdimensions ranged from 2.142 to 4.340, with support behavior scoring the highest, followed by collegial, intimate, directive, restrictive, and disengaged behavior.

**TABLE 1:** Descriptive results of the overall level of transformational leadership and organizational climate in kindergarten.

	***M***	***SD***
Transformational leadership	4.431	0.410
Visionary	4.194	0.653
Charisma	4.315	0.510
Individualized consideration	4.436	0.527
Morale modeling	4.779	0.349
Organizational climate	3.299	0.246
Support behavior	4.340	0.527
Directive behavior	2.912	0.552
Restrictive behavior	2.201	0.653
Collegial behavior	4.120	0.573
Intimate behavior	4.078	0.525
Disengaged behavior	2.142	0.607

#### LCA of the Transformational Leadership of Kindergarten Principals

Using a single-class model as a baseline, one category at a time was added to determine the minimum number of latent classes that would accurately model the relationship between the explicit behavior indicators of transformational leadership. Table 2 lists the fit indices found for a single-class model (C1) to a five-class model (C5). In the single-class model, the LMR and BLRT values were not significant, and the AIC, BIC, and aBIC values were relatively large. In the two-category model, the AIC, BIC, and aBIC values decreased gradually, the entropy value was 0.9, and the LMR and BLRT were significant. With three categories, the AIC, BIC, and aBIC values were smaller, the entropy value was larger, and the LMR and BLRT were significant. As the number of categories increased, AIC, BIC, and aBIC decreased, and entropy reached a maximum in the four-category model, but LMR was not significant, and BIC increased (see Table 2). Therefore, it was most reasonable to consider a three-category model, within which the class probabilities of C1, C2, and C3 were 0.705, 0.243, and 0.052, respectively. Table 3 shows the attribution probability matrix for three latent classes. According to Table 3, the average probability of each class attributable to each latent class is between 95.7% and 100%, indicating that the results of the three-latent-class model are reliable.

**TABLE 2:** The LCA fit index of transformational leadership.

**Models**	***K***	**Log(L)**	**AIC**	**BIC**	**aBIC**	**Entropy**	**LMR**	**BLRT**	**Class probability**
1	22	−3041.15	6126.298	6218.932	6149.102	–	–	–	
2	45	−2533.85	5157.689	5347.166	5204.334	0.9	^∗∗∗^	^∗∗∗^	0.763/0.237
3	68	−2436.77	5009.547	5295.868	5080.033	0.911	^∗^	^∗∗∗^	0.705/0.243/0.052
4	91	−2380.29	4942.577	5325.742	5036.904	0.912	0.119	^∗∗∗^	0.694/0.199/0.054/0.052
5	114	−2338.02	4904.041	5384.05	5022.209	0.912	0.211	^∗∗∗^	0.108/0.052/0.648/0.035

*K, number of parameters estimated freely; – No such value; ^∗^*p* < 0.05, ^∗∗^*p* < 0.01, ^∗∗∗^*p* < 0.001.*

**TABLE 3:** Average attributable probability of participants (rows) for each latent category (column).

**Classes**	**C1 (%)**	**C2 (%)**	**C3 (%)**
C1	1.000	0.038	0.000
C2	0.000	0.957	0.003
C3	0.000	0.010	0.990

**FIGURE 1:**
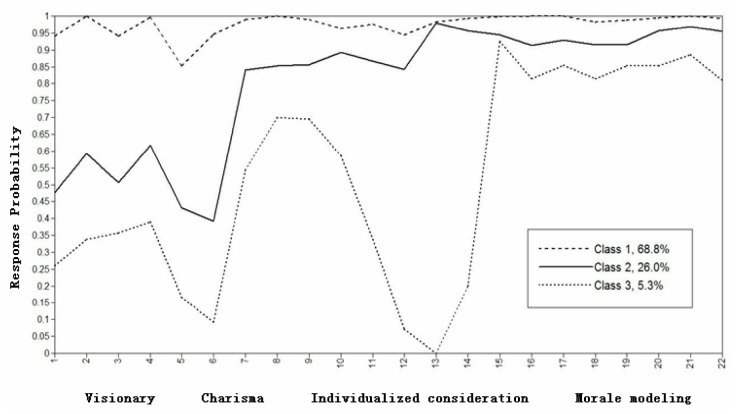
The response probability diagram of each latent class on all entries. In data analysis, the original scale items are rearranged according to the items included in each dimension, i.e., 1∼4 is the visionary, 5∼9 is the charisma. 10∼14 is the individualized consideration. 15∼22 is the morale modeling.

Based on the LCA, the response probability diagram of the three latent categories on 22 entries was obtained, as shown in Figure 1. According to the response probability characteristics, C1–C3 were given distinct titles. Figure 1 demonstrates that the proportion of group C1 was 68.8%. In this group, the conditional probability was higher across the four dimensions than it was in the other two groups; thus, it was named the “high-level group.” The proportion of C2 group was 35.7%. The conditional probability of the vision dimension was moderate, and the overall conditional probability level of charisma was high; however, the conditional probability of the fifth and sixth items within this dimension was lower. The fifth item was “strong business ability”; the sixth item was “enlightened, with a strong sense of innovation.” Finally, conditional probability in the dimensions of individual consideration and moral modeling was high. The principals classified within this category were notably characterized by high moral quality and care for subordinates, but their professional ability levels were low, and they could not effectively lead subordinates to make efforts toward a given goal. Therefore, this group was named the “care-virtues group.” The proportion of C3 group was 5.3%. Conditional probability was only high in the moral dimension, so this group was called the “virtues group.”

### Comparison of the Organizational Climate of Kindergartens With Different Leadership Types

Taking each latent category as an independent variable and organizational climate factors as dependent variables, the results indicated significant differences in support behavior, collegial behavior, intimate behavior, directive behavior, restrictive behavior, and disengaged behavior between kindergartens with different leadership types, as shown in Table 4. After the *post hoc* tests, it was found that the high-level group had the highest scores in support behavior, collegial behavior, and intimate behavior, followed by the care-virtues group and the virtues group, respectively. Regarding restrictive behavior and disengaged behavior, the virtues group had the highest scores, followed by the care-virtues group and the high-level group. According to the estimated effect size, the strongest relationship existed between the transformational leadership classes and collegial behavior, followed by the relationships with support, intimate, disengaged, and restrictive behavior, respectively.

**TABLE 4:** Comparison of organizational climate scores of kindergartens with different leadership types (M ± SD).

	**The type of transformational**							
	**leadership of the director**							
	**of the kindergarten**							
						***Post hoc* tests (*P*)**		
	**The**	**The**	**The**					
	**high-level**	**care-virtues**	**virtues**			**①**	**①**	**②**
	**group ①**	**group ②**	**group ③**					
**Variables**	**(*n* = 351)**	**(*n* = 121)**	**(*n* = 26)**	***F***	**η^2^**	②	③	③
Supportive behavior	4.4 ± 0.5	4.2 ± 0.5	3.7 ± 0.5	36.40^∗∗∗^	0.128	*p* < 0.001	*p* < 0.001	*p* < 0.001
Directive behavior	2.9 ± 0.6	2.9 ± 0.5	3.0 ± 0.5	0.58	0.002	1.000	0.963	1.000
Restrictive behavior	2.1 ± 0.6	2.3 ± 0.6	2.7 ± 0.8	9.76^∗∗∗^	0.038	0.042	*p* < 0.001	0.036
Colleague behavior	4.3 ± 0.5	3.8 ± 0.6	3.5 ± 0.6	52.97^∗∗∗^	0.176	*p* < 0.001	*p* < 0.001	0.007
Intimate behavior	4.2 ± 0.5	3.9 ± 0.5	3.5 ± 0.5	34.54^∗∗∗^	0.122	*p* < 0.001	*p* < 0.001	*p* < 0.001
Disengaged behavior	2.1 ± 0.6	2.3 ± 0.6	2.5 ± 0.6	13.35^∗∗∗^	0.051	*p* < 0.001	*p* < 0.001	0.179

*^∗∗∗^*p* < 0.001.*

## Discussion

### Transformational Leadership Status of Kindergarten Directors

This study showed that principals’ overall leadership levels and the scores in each subdimension were above average (3 points, which was available to the respondents), meaning that the transformational leadership of kindergarten principals was generally at a high level, which is consistent with Liu’s (2015) findings. This study also used a questionnaire survey method that took the kindergarten director as the study object to investigate the leadership level of the director, and it found that the overall leadership levels of the directors were high. This may be because the same questionnaire was used, and the same dimensions were measured. It could also be attributable to the complexity of the work itself, which might require high levels of leadership from kindergarten directors. Thus, attaining a position as a principal may be indicative of high levels of leadership. Across groups in this study, scores in each dimension ranked as follows, from high to low: moral modeling, individual consideration, charisma, and vision. This demonstrates that directors display high levels of political and moral qualities but may not be thorough in their planning for the development of kindergartens. Therefore, their vision scores are low. One explanation for this result may be the influence of moral norms in a traditional culture. Morality exerts a subtle influence on individuals. As leaders, kindergarten principals may naturally improve their own moral standards. In addition, the country has vigorously advocated socialist values, which may have encouraged directors to improve. Regarding the development of kindergartens, principals’ overall understanding of organizational culture may not be deep enough, and principals may be unaware of the importance of defining common goals and promoting cohesion. Further, it is very likely that directors have few opportunities to learn about kindergarten management, as they are often self-appointed and lack motivation for active learning.

This study found that overall, the organizational climate in kindergartens is moderately positive and requires further improvement. Factors positively effecting climate, including support, collegial, and intimate behavior, scored higher than did negative factors, including directive behavior, restrictive behavior, and disengaged behavior. Disengaged behavior received the lowest score, which is in line with Wang’s (2010) results. Through the questionnaire survey, it was also found that the average score of positive organizational climate factors was higher than that of negative organizational factors, and the score of teachers’ disengaged behavior was the lowest. This suggests that the formation of cliques or turnover among kindergarten teachers is less common than other behaviors. This may be because most kindergarten teachers have a positive attitude toward their work, and the relationship between teachers is harmonious, leading to a positive overall climate. Thus, the results demonstrate that in kindergartens, the relationship between directors and teachers is often positive. Both the directors’ and the teachers’ professions require patience and love. Therefore, the organizational climate in kindergarten benefits from these qualities. Demographic variables did not significantly affect kindergarten organizational climates, but principals’ leadership levels were closely related with the organizational climates of kindergartens.

### LCA of Principals’ Transformational Leadership

This study’s results show that the leadership of kindergarten directors has obvious classification characteristics, and the indicators of each of the three potential categories show good adaptability.

Among them, the response probability of the high-level group was higher across all dimensions. The group’s average scores in vision, charisma, individual consideration, and moral modeling were relatively high, indicating strong leadership. The care-virtues group had moderate scores in the vision and charisma dimension, while the conditional probability of individual consideration and moral modeling dimensions was high. The virtues group had low scores in the vision and charisma dimensions and particularly in the individual consideration dimension, but the group’s level of moral modeling was relatively high. Individual consideration plays an important role in transformational leadership. If new employees are provided with life and work assistance, they will be more willing to follow a leader in completing a task (Li, 2014). “If you see a pawn like a baby, you can go deep with it; If you see a pawn like a son, you can die with it.” Therefore, principals belonging to the virtues group should improve their ability to listen to teachers’ feelings and ideas, pay unconditional and active attention to teachers, have positive interactions with them, and try to view problems from the teachers’ perspectives. As principals in all groups scored relatively high in moral modeling, it seems that they are likely to attach great importance to their moral influence within the kindergarten and behave accordingly.

### Principles’ Transformational Leadership and Organizational Climate

According to the variance analysis of the three types of transformational leadership among principals, the virtues group received the lowest score regarding organizational climate, followed by the care-virtues group and the high-level group, respectively. This is consistent with the hypothesis that the directors’ leadership can predict kindergartens’ organization climates. This reflects the director’s important role in the kindergarten, especially in leading the kindergarten teachers. If principals possess high professional abilities, moral quality, and levels of care for teachers and actively lead the development of the kindergarten, teachers and the director can share a sense of cohesion and form a positive organizational climate. Members of the virtues group received very low ratings in individual consideration and low ratings for vision and charisma. Such principals have relatively weak abilities, and their interactions with subordinates are mostly work related; they rarely care about their employees’ personal lives. Therefore, teachers do not feel support and concern from such directors, and the kindergartens under their leadership do not form organizational values or shared visions among teachers, parents, and children. Thus, teachers simply face their daily work and have no expectation of their kindergartens or of themselves. They can easily become alienated from their work environments, which, in turn, affects the formation of a positive organizational atmosphere in kindergartens. Thus, directors should listen carefully to the individual needs of the members of the organization, empower teachers to meet challenges, and care for teachers in a positive and personal manner, helping teachers develop a strong sense of belonging, actively participate in the achievement of objectives, and improve their performance. Raising principals’ levels of transformational leadership is an effective way to establish positive organizational atmospheres in kindergartens. The relevant departments of the state should attach great importance to training and providing financial and policy support to kindergarten directors. Directors should improve their professional skills, care for teachers, and actively build a shared vision with teachers and parents. Finally, researchers should actively explore effective management practices.

## Conclusion

This study mainly discussed the classification characteristics of kindergarten directors’ leadership and the relationship of directors’ leadership with kindergarten organizational climate. The analysis of latent categories identified three latent categories of transformational leadership: the high-level group (68.8%), the care-virtues group (35.7%), and the virtues group (5.3%). Kindergartens’ organizational climate is affected by different types of leadership, and there are significant differences in support, directive, restrictive, collegial, intimate, and disengaged behavior among different climates. Regarding support, collegial, and intimate behavior, directors’ leadership scores were highest in the high-level group, followed by the care-virtues group and the virtues group. Regarding restrictive and disengaged behavior, scores were the highest with the virtues group, followed by the care-virtues group and the high-level group. The study confirmed that directors’ leadership styles had obvious classification characteristics and can predict the organizational climate within kindergartens. This study used the latent category analysis method to explore the leadership styles of kindergarten directors, thus expanding the research on kindergarten directors’ leadership. This study’s results offer specific guidance for leadership training for principals, which could make future training more targeted. Future research could further explore the differences in the organizational climate and management of kindergartens according to the different leadership styles of kindergarten principals through empirical research, thus promoting the development of kindergartens and improving the quality of education afforded to children.

## Limitations

This study is not without limitations. These include that all conclusions are based on a specific economic background and specific geographical area, specifically those in China.

This limits the generalizability of these results, because people from different cultural backgrounds may not hold the same beliefs about kindergarten principals’ transformational leadership. Thus, future research can compensate for this deficiency, and we welcome researchers from different cultural and economic backgrounds and different regions to replicate this study. Another limitation is that data were generated through the self-reporting of principals. Future studies could be improved by examining teachers’ reports.

## Data Availability

The datasets generated for this study are available on request to the corresponding author.

## Ethics Statement

The studies involving human participants were reviewed and approved by the Academic Ethics Committee of the College of Psychology of Northeast Normal University. Written informed consent for participation was not required for this study in accordance with the national legislation and the institutional requirements.

## Author Contributions

PW wrote and modified the manuscript. XD, XL, and YD collected and collated the data. RJ recruited the participants.

## Conflict of Interest Statement

The authors declare that the research was conducted in the absence of any commercial or financial relationships that could be construed as a potential conflict of interest.
